# Piezoelectric Bone Surgery: A Review of the Literature and Potential Applications in Veterinary Oromaxillofacial Surgery

**DOI:** 10.3389/fvets.2015.00008

**Published:** 2015-05-05

**Authors:** Philippe Hennet

**Affiliations:** ^1^ADVETIA Veterinary Specialty Center, Paris, France

**Keywords:** piezoelectric, ultrasonic, maxillofacial, surgery, dog, cats

## Abstract

Piezoelectric bone surgery is a recent and innovative technology, permitting a selective cut of mineralized tissue while sparing soft tissue. Similar to a dental scaler, a high frequency vibration, in the range of 25–35 kHz, is transmitted to a metallic tip. However, the power of the piezosurgical instrument is three to six times higher than that of a dental scaler. The major advantages of this technology include high precision, a design that increases ease of curvilinear osteotomy, less trauma to soft tissue, preservation of neurological and vascular structures, reduced hemorrhage, minimal thermal damage to the bone, as well as overall improvement of healing. The handpiece of the instrument is equipped with a sterile irrigation system and light-emitting diode (LED) light, which improves visibility and overall safety. Piezoelectric surgery is particularly useful when performing delicate bone procedures such as periodontal or endodontic surgery. It is also indicated when performing more invasive bone surgery such as maxillectomy, mandibulectomy, and condylectomy, where preservation of neurovascular structures is important. Piezoelectric instruments are different from rotary instrumentation or oscillating saws, they require light pressure with constant motion of the tip. Training is required to master the technique.

Oral and maxillofacial surgery often involves bone surgery. These can be either to prepare or permit access to the surgical site or as part of a radical resection in oncology. Bone cutting can be performed with different instruments, either manual (bone chisels, rongeurs) or powered (rotary burs, oscillating saws). Manual bone cutting instruments do not generate heat but require use of high forces, which may lead to uncontrolled damage to the bone or to the surrounding structures. Use of such high forces is not necessary with rotary drills and oscillating saws. However, heat is produced during cutting and the degree of thermal injury to the tissues is related to parameters of the instrument (design, diameter, sharpness, material) and to parameters of bone cutting (speed, feed rate, power, depth of cut, cooling, clearance of bone chips, bone density, and thickness) (1–4). Physical or thermal injury to the bone may result in cell death, lack of regeneration, and bone lysis (1, 5). Furthermore, powered instruments may entrap surrounding soft tissues during osteotomy resulting in severe damage to muscles, nerves, and blood vessels especially at sites with difficult or limited accesses. Piezoelectric bone surgery is a technology based on the high frequency vibration of a metallic tip used to selectively cut bone while sparing surrounding soft tissues. Ultrasonic bone cutting instruments were first proposed by Vang in 1955; however, they did not gain popularity until the early 21st century. This coincided with the need for more precise osteotomies as the emphasis in maxillofacial surgery extended to involve cortical bone graft collection and surgery of sinus in preparation for dental implant placement (6, 7). The technology is based on inverse piezoelectric activity: alternative current applied to piezoactive ceramic disks generates high-frequency vibratory energy (8). Frequencies of 25–35 kHz (Hertz = vibrations/s) are specific for cutting mineralized tissue, whereas soft tissue incisions require frequencies above 50 kHz (7, 9). The vibrations generated by the ceramic pellets contained in the handle of the instruments induce a high-frequency almost linear reciprocal motion of the metallic tip of the instrument resulting in up to 300 μm excursions (9, 10). The piezoelectric bone surgery unit vibrates at a similar frequency to the ultrasonic piezoelectric dental scaler, however, it is three to six times more powerful (11).

## Advantages of Piezoelectric Bone Surgery

### Selective cut of mineralized tissue

At frequencies of 25–35 kHz, the instrument is only cutting mineralized tissue (Table 1). Soft tissue vibrates without rupture at the same frequency as the tip of the instrument, trauma of vascular or neurological structures are dramatically reduced (7, 9, 12). Nevertheless, soft tissue damage may occur when the structure is tightly entrapped or bound to the bone and, subsequently, cannot freely vibrate.

**Table 1:** **Advantages of piezoelectric surgery**.

• Selective cutting of mineralized tissue
• Significant reduction of trauma to soft tissue
• Reduced hemorrhage (cavitation effect)
• Excellent visibility within the surgical field, due in part to minimal bleeding, to high luminosity LED lights and effective irrigation
• Precise cutting (limited vibration amplitude and specific design of osteotome tips)
• Curvilinear cutting
• No thermal damage
• Sterile irrigation – steam sterilization

### Cutting power

The latest piezoelectric bone surgery instruments have an increased power (up to 90 W) compared to first generation instruments (5–15 W). A higher power has been claimed to enhance bone-cutting efficacy in hard bone (13, 14). However, no study comparing power and cutting efficacy of piezoelectric devices could be identified in the literature. It is not clear whether the power claimed by manufacturer relates to the electric power supplied by the ultrasonic generator or to the vibratory power transmitted to the probe-tissue interface and resulting in surgical cutting. In an experimental model on bovine femur shafts, a unit with 50 W power performed less efficiently than two other units with respective powers of 16 and 55 W (5). In the latter study, cutting rates of 0.25 mm/s and of 0.3 mm/s were respectively observed with the Piezosurgery II^®^ unit (Mectron, Carasco, Italy) and the Piezotome2^®^ unit (Satelec, Mérignac, France) when used at maximum power (respectively 16 and 55 W) with a irrigation of 50 ml/s (5). The cutting power is influenced by bone density, tip characteristics (alloy, shape, sharpness), and working pressure (7, 9). Contrary to rotary instruments and oscillating saws, the piezoelectric tips do not require pressure on bone to be effective and, therefore, thermal injuries and bone microfractures are reduced (6, 9, 15, 16). It is generally accepted that cutting with piezoelectric instruments takes longer than with rotary burs or oscillating saws, especially when the surgeon is learning this technology (6, 10, 15, 17, 18). Spinelli et al. showed in a recent maxillofacial study in humans that the whole surgical procedure took 35% longer with a piezoelectric bone unit compared with an oscillating saw (10). However, there are important variations from one study to another depending on the type of surgery, the type of instrument, and the experience of the surgeon. In a randomized clinical evaluation of piezoelectric surgery versus rotary burs for lower third molar extractions, Barone et al. showed that piezoelectric bone surgery was faster (19). In a recent *in vivo* study on ventral slot surgery in dogs, mean surgical duration for piezoelectric intruments was significantly shorter than for surgical burs (23.4 versus 34.1 min) (20). Cutting efficiency of piezoelectric instrument has also been compared with that of laser Er:YAG in an experimental study on sheep tibia; time necessary for cutting a 18 mm × 22 mm bone segment at mid-shaft was 160–200 s with the piezoelectric instrument and longer with the laser (21).

### Safety

#### Reduced Neurological Trauma

It has been shown that a piezoelectric surgical instrument directly applied for 5 s on a peripheral nerve with a relatively high working force (1.5 N) did not dissect the nerve but induced some structural and functional damage (22). The perineurium of the nerve remained intact even after nerve contact at peak force (3 N), thus enhancing the potential for functional recovery. Importantly, the extent of damage was significantly higher with application of increased force on the nerve by the device, but not by activation of the ultrasonic vibration (22). The efficacy and safety of piezoelectric bone surgery has been assessed in neurosurgery where it has been shown that osteotomy of parietal bone of the cranial vault could be achieved without trauma to the dura matter (16).

#### Reduced Bleeding

Less bleeding is observed during maxillofacial surgery with piezoelectric instruments compared to surgical burs or oscillating saws; blood loss is reduced by 25–30% (10, 17). There are different factors responsible for the reduction in blood loss. Due to a better visibility and to the sparing-effect on soft tissues, blood vessel damage is reduced. Furthermore, cavitation effect associated with ultrasonic instrumentation has been shown to be responsible for the relative absence of blood at the surgical site (17, 23). Microbubbles of gas dissolved in the fluid expand and contract when exposed to sinusoidal ultrasound waves. When they reach a resonant size, the microbubbles violently collapse creating a shock wave (6).

### Reduced tissue trauma and improved healing

A significant reduction in postoperative edema and hematoma has been observed when maxillofacial surgery is performed with piezoelectric instruments compared to an oscillating saw (10). In a study comparing human patients that had undergone mandibular third molar extractions with a piezoelectric instrument to those that had the same procedure performed using a surgical drill, it was found that the piezoelectric group had less postoperative inflammation, trismus, and pain, as well as a reduced necessity for postoperative medications (19). An experimental study on bovine cortical bone has compared intraosseous temperatures induced by different piezoelectric instruments under controlled conditions. When the instruments were used at maximum power, irrigation at 50 ml/s and cutting pressure of 3 N, mean intraosseous temperature increases between 1.2 and 3.1°C were observed, with Piezotome2^®^ (Satelec, Mérignac, France) producing the less heat (5). The temperature reached within bone was well below critical levels for intraosseous hyperthermia (40–41°C) and bone necrosis (47°C) (24). The degree of the thermal bone damage is directly proportional to the resulting temperature at the cutting site and the period of time the bone is exposed to that temperature. Under clinical conditions, a smaller amount of irrigant may be used or may not reach the tip of the instrument in deep cuts and may result in higher intraosseous temperatures. Brief temperature rises above 47°C might not be detrimental if shorter than a few seconds (24). To lessen the risk of thermal damages, continuous motion and pressure on bone should be avoided especially in deep cuts (11, 25). Intermittent motion applied to the bone with light pressure and copious irrigation helps preventing heat build-up (24).

### User friendly and precision

**Figure 1:**
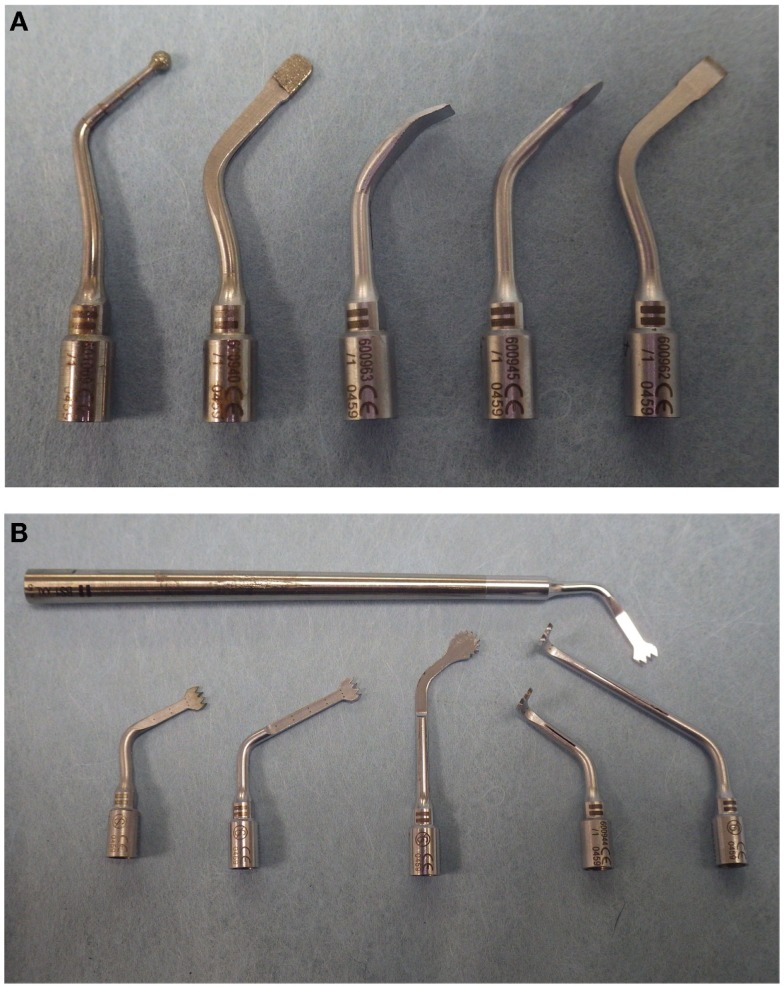
**Various tips for Piezotome2^®^ (Satelec, France)**. **(A)**: Diamond-coated and scalpel piezoelectric tips for Piezotome2^®^ (Satelec, France). From left to right, diamond-coated round tip, diamond-coated flat tip, square scraper, rounded scraper, flat chisel. **(B)**: Serrated tips are available in different lengths of blade or shank; the head of the tip may be straight, round or angled at 90° to facilitate access to a variety of surgical sites.

Several tip attachments are available, these differ in shapes, length, and design so as to adapt to specific anatomic regions or types of surgery. The tips are grouped by basic shape: sharp (scalpels and scrapers), serrated (saw), or diamond-coated (Figures 1A,B). Because of the various tips, their small size as well as the cutting technology, the instrument can create a precise and thin cutting line in any direction even in areas with a difficult access.

When using a surgical bur or saw, the cutting efficiency is linked to the pressure on bone. With a piezoelectric unit, cutting is due to the high frequency vibration of the tip of the instrument; excessive pressure prevents vibration, decreases efficiency, and generates frictional heat. The handle of the instruments is held with a modified pen grasp. A moderate force (1.5 –3 N) is used to allow the tip to vibrate (5, 22, 26–28). As a point of comparison, the axial force of handwriting has been reported to be close to 1 N (29, 30). A Working pressure of 1.5 and 2.0 N with a minimum of 30 ml/min cooling irrigation has been shown to fulfill the requirements for harmless intraosseous temperature. Beyond 3 N, cutting efficiency was not improved but thermal damage was increased (27). When the tip contacts cortical bone, then it quickly produces an increase in depth from loads of 50 to 100 g, but there is a decrease at 200 g, which mirrors the decrease in displacement amplitude (28). A load of 150 g (approximately 1.5 N) has been therefore recommended for cutting cortical bone (26, 28). It is obvious from previous studies that the optimum force required to cut bone depends on the type of bone (cortical or spongious), its density (degree of mineralization), and the type of ultrasonic tip used (5, 25–28, 31). The surgeon must therefore get used to the cutting action of these new instruments and develop a tactile sense of how much pressure needs to be used in the different clinical situations.

Because this technology is recent and there have been very few studies on the cutting effect of the different tips on various bone types, clinical use is based more on personal experience and manufacturer’s recommendations than on scientific evaluations of the cutting performance of these instruments (26). When performing an osteotomy on dense cortical bone, the more powerful sharp serrated (saw-like) tips can be used. The tip is held perpendicular to the bone surface, and with copious irrigation (50 ml/s), the surface of the bone is penetrated with the tip in a press cut action to form a punctuated line. Then, the tip is moved back and forth along this punctuated line to create a groove. In more difficult areas, the bone can be cut to full depth using a press cut action. In deep cuts, care must be taken that the irrigation fluid reaches the tip and intermittent motions are used. The tip should not be angled laterally during the cutting to avoid breakage, it must be kept at right angle to the surface. The handpiece is equipped with a sterile irrigation system. This helps to clear the surgical field, clean bone chips, dissipate heat due to friction, and improve hemostasis through the cavitation effect. The handpiece is equipped with high luminosity LEDs (100,000 Lux), which improves visibility at the surgical site even in deep locations.

## Limitations

### Speed of osteotomy

As seen before, piezoelectric bone surgery is usually considered to be slower than osteotomy with a bur or with an oscillating saw (6, 10, 15, 18). Nevertheless, constant improvement of this new technology by manufacturers has led to more efficient instruments, which with the learning curve by the surgeon, minimizes this problem (17, 18, 32).

### Learning phase

Piezoelectric surgery is associated with an initial learning curve. It takes time to learn how to apply minimal pressure so as to allow the tip to vibrate effectively during the osteotomy (10, 18, 32).

### Cost

The cost of piezoelectric bone surgery units ranges from 5000 to 7000 euros depending on the brand, the specifications, and equipment included. Replacement of a piezoelectric tip ranges from 150 to 200 euros/tip.

## Clinical Uses

There are many uses for piezoelectric surgery in dentistry and in oromaxillofacial surgery, owing to the various and versatile tips that can be adapted on the handle of the instrument (Table 2). They enable the surgeon to perform osteoplasty and osteotomy procedure on different bone densities as well as to harvest bone by scraping (33). Diamond-coated tips are cutting by abrasion and therefore may be considered less aggressive and safer on thin bone or in the vicinity of soft tissue; there are commonly used for sinus surgery in order to minimize the risk of mucosal tear (11, 25). Flat sharp tips (scalpel-like) are intermediate and are usually used to scrape cortical bone, to cut bone, or to perform osteoplasty.

**Table 2:** **Indications of piezobone surgery in OMF**.

• Dentistry
○ Periodontal bone surgery
○ Apicoectomy
• Oral surgery
○ Ostectomy and osteoplasty
○ Surgical extractions
○ Extraction of impacted teeth
○ Root tip extraction
○ Harvesting of cortical bone grafts
• Maxillofacial surgery
○ Segmental mandibulectomy and rim excision
○ Maxillectomy
○ Orbitectomy
○ Coronoid process resection
○ TMJ surgery
• ENT surgery
○ Dorsal and ventral rhinotomy
○ Tympanic bulla osteotomy and curettage

Though originally developed for implantology and maxillofacial surgery, piezoelectric surgery is nowadays used in many fields such as ear nose and throat surgery, neurosurgery, and orthopedic surgery in humans (8, 9, 11, 12). There are very few publications on the use of piezoelectric bone surgery in the veterinary field (7, 20, 34). Based on personal experience, most common indications include periodontal bone surgery, complex exodontics (impacted teeth), apical surgical access and apical resection, mandibulectomy and maxillectomy, cortical bone graft harvesting for reconstructive procedures, zygomatic arch resection, temporomandibular joint (TMJ) resection, difficult dental extractions, osteotomy associated with dorsal or ventral rhinotomy, as well as tympanic bulla osteotomy and curettage in ear surgery (Table 2). This instrumentation may also find uses in other veterinary fields such as surgery of the cranium, spinal surgery, and orthopedics (7, 20).

### Exodontics

Although most surgical tooth extraction procedures can be performed using traditional methods (rotary and hand instruments), piezoelectric tips can also be used for tooth sectioning, bone removal and root luxation. In the author’s experience, piezoelectric tips do not offer a clear advantage as far as operative speed when performing routine surgical extractions. However, they were found very useful in certain situations such as extraction of impacted teeth, retrieval of root tips, and dental extractions in animals with limited jaw opening consecutive to TMJ or muscular disorders. They are specific piezoelectric tips designed by manufacturers for that use.

### Periodontal bone surgery

The author has been using piezoelectric instruments to perform ostectomy and osteoplasty associated with apically repositioned flaps and to harvest autologous bone graft. Sharp tips (scalpel-like) can be used to perform bone cutting as well as bone scraping depending on their design (flat or curved). The head of the tip can be rounded or straight. They allow a very sharp and precise cut under sterile irrigation minimizing any thermal damage to bone cells. This is a critical issue. When gentle bone removal or osteoplasty is needed, another option is the use of diamond coated-tips. They are commonly used in humans to cut a window into the sinus wall, while sparing the sinus mucosa is therefore considered very safe in contact with soft tissue (11, 25, 35). They can be used with a pen-like motion or with a swamping motion on bone surface to cut or remodel bone.

### Endodontic surgery

**Figure 2:**
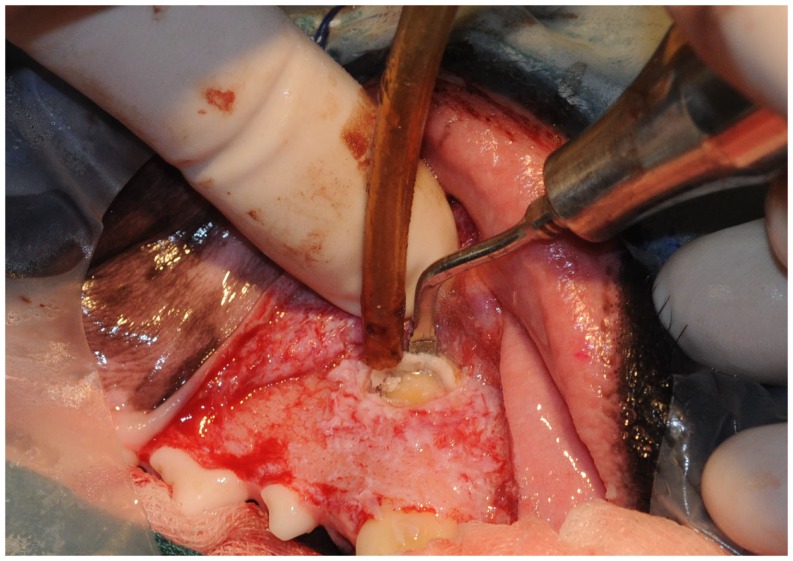
**Bone trephination to expose the apex of the right maxillary canine tooth for endodontic surgery**.

Piezoelectric bone cutting tips can be used both for bone fenestration to expose the apex of a tooth as well as for the performance of an apicoectomy (36). On the maxilla, apex exposure can be easily achieved by removing the thin cortical bone with a curved scalpel-like tip used as a bone scraper, or with a diamond-coated tip. On the mandible, when using a ventral cutaneous approach to the apex, the cortical bone is denser and thicker. The author had better success using a more powerful serrated tip or a sharp flat scalpel-like tip. Apical resection of the root can be performed with the same type of tips. The cut surface of the root can be further smoothed and flattened with a flat diamond-coated tip used in a swamping motion. Finally, apical root canal preparation being nowadays performed with endodontic ultrasonic tips, the whole procedure can be completed with piezoelectric instruments solely with no further use of burs (Figure 2).

### Mandibulectomy

**Figure 3:**
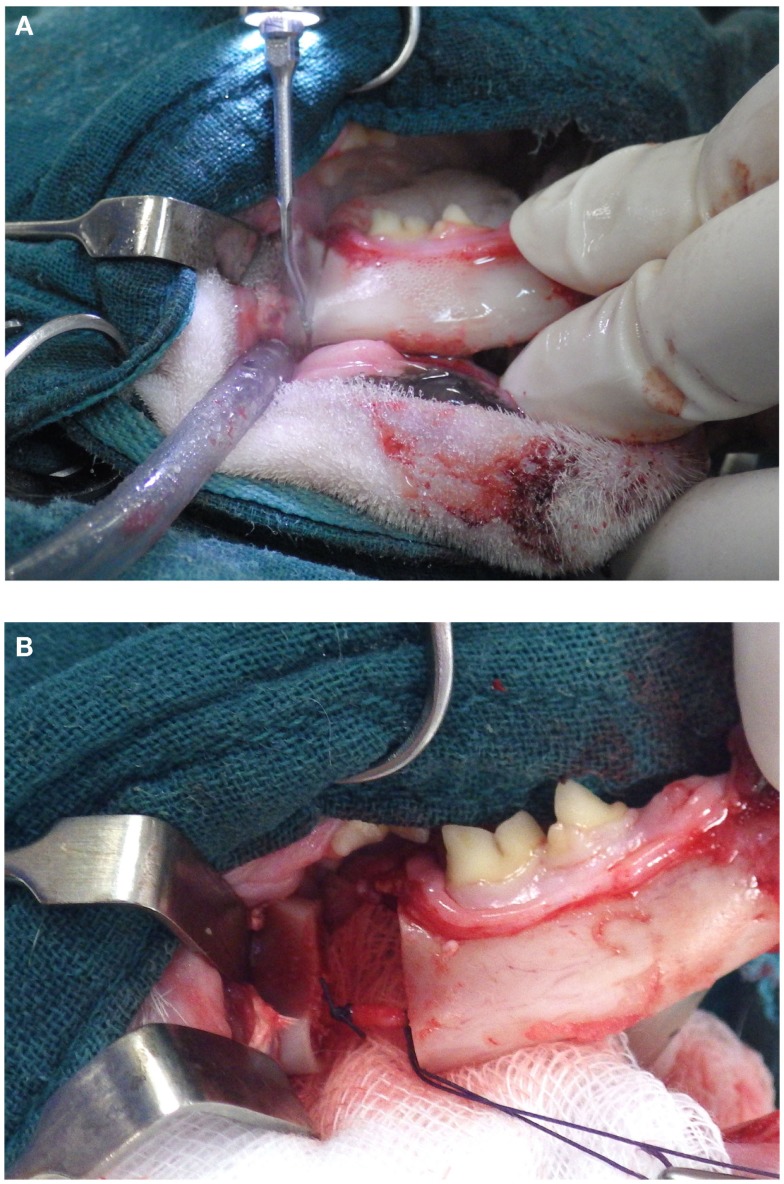
**(A, B)** Osteotomy of the body of the mandible without sectioning the neurovascular bundle. It can be properly ligated and sectioned.

Piezoelectric surgery allows the cutting of the mandibular cortex without severing the neurovascular bundle within the mandibular canal. The author preferentially uses serrated tip for cutting the dense cortical bone when performing mandibulectomy. Nevertheless, care should be taken not to go through the whole thickness of the body of the mandible in the location of the mandibular canal but rather to cut the cortical bone on its periphery. Direct contact of the piezoelectric tip with the nerve or vessels does not directly induce damages unless uncontrolled and excessive pressure against theses structures is exerted. Once the osteotomy of body of the mandible is performed, the rostral and caudal parts can be pulled apart to expose the blood vessels and nerve, which can be ligated and cut (Figures 3A,B).

### Maxillectomy

**Figure 4:**
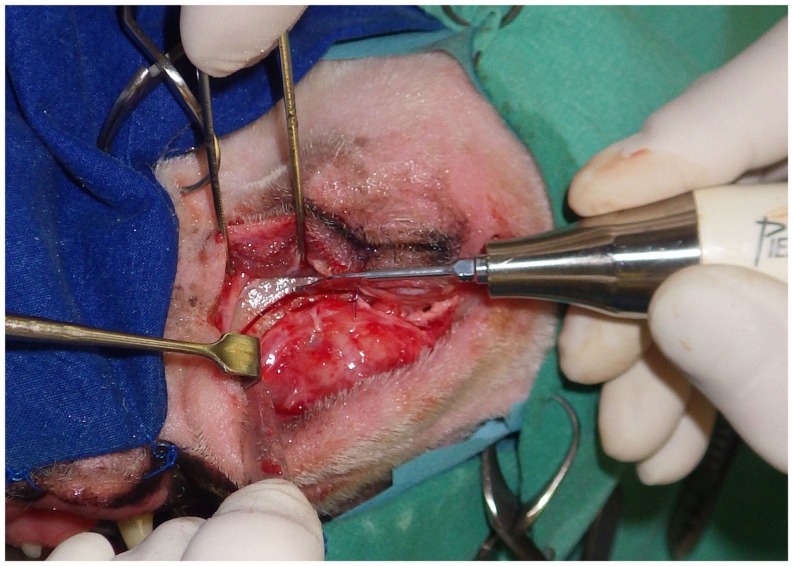
**Caudal maxillectomy through a cutaneous approach below the eye**. Piezoelectric instruments can create various osteotomy lines, causes less bleeding, and are safer than a rotary bur or than an oscillating saw in such a complex surgical site.

**Figure 5:**
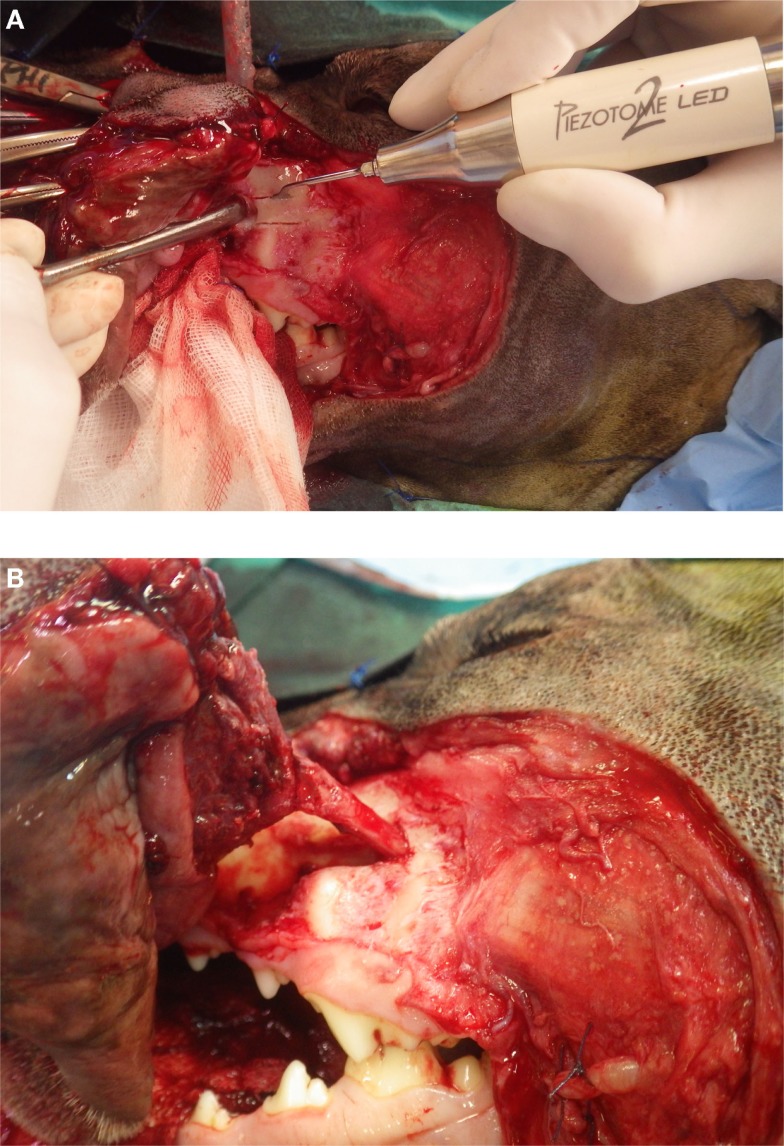
**(A, B)** Opening of the infraorbital canal to isolate the neurovascular bundle permitting resection of the cheek tumor with adequate margins.

Maxillectomy, especially in the caudal part of the jaw, is associated with a more complex anatomy. Jaw resection may extend into the nasal cavity and in the orbital area where major arteries and nerves are located (Figure 4). Piezoelectric bone surgery is the technique of choice in these difficult surgical cases, bleeding is reduced, risk of damage to blood vessels coming from the nasal cavity and orbital area is limited, and the osteotomy line can be adapted to both the surgical margins and anatomical structures. Additionally, repositioning of the neurovascular bundle can be safely performed by opening the mandibular or the infraorbital canal with a piezoelectric tip. This might be useful when resection of the nerve or of the neurovascular bundle at the site of osteotomy is not necessary but would only be dictated by the impossibility to spare it while doing osteotomy with conventional instruments (Figures 5A,B).

### Temporomandibular joint surgery

Temporomandibular joint ankylosis is the main indication for TMJ surgery in small animals, especially in cats. Condylectomy is a delicate procedure as the surgical access is narrow, the condylar process is elongated latero-medially, and the maxillary artery is located medial to the joint. When facing TMJ ankylosis, the normal anatomy is often distorted with severe bone remodeling and fusion. The author has had experience of power and hand instruments (chisel and rongeurs) prior to piezoelectric instruments for performing condylectomy. In his opinion, piezoelectric bone cutting instruments offer major advantage when dealing with this complex osteotomy. The LED illumination, continuous irrigation, precise cutting, and preservation of vascular structures allow a more effective and safer surgical procedure. Recently, this advantage was documented in the treatment of TMJ ankylosis in humans (37).

## Conclusion

Piezoelectric bone surgery constitutes a new and exciting field in veterinary oral and maxillofacial surgery. It provides major advantages over hand instruments, a surgical bur, or an oscillating saw in a surgical field with limited access or close to major neurovascular structures. Piezoelectric bone cutting does not require a high force reducing the risks of collateral damage. The selective cutting of mineralized tissue, the sparing effect on neurovascular structures, the precision with which curvilinear osteotomy lines can be performed, the reduction in bleeding, as well as the increased visibility due to the sterile irrigation system, and the high luminosity LED lights largely overcome a slightly increased cutting time compared to other instruments.

## Conflict of Interest Statement

The author declares that the research was conducted in the absence of any commercial or financial relationships that could be construed as a potential conflict of interest.
